# Recurrence and mortality after non-operative management of acute gallstone disease: nationwide registry study

**DOI:** 10.1093/bjsopen/zrag149

**Published:** 2026-11-04

**Authors:** Maria Söderström, Olov Norlén, Fredrik Linder, Erik Osterman

**Affiliations:** Department of Surgical Sciences, Uppsala University, Uppsala, Sweden; Department of Surgery, Akademiska Sjukhuset, Uppsala, Sweden; Department of Surgical Sciences, Uppsala University, Uppsala, Sweden; Department of Surgery, Akademiska Sjukhuset, Uppsala, Sweden; Department of Surgical Sciences, Uppsala University, Uppsala, Sweden; Department of Surgery, Akademiska Sjukhuset, Uppsala, Sweden; Department of Surgical Sciences, Uppsala University, Uppsala, Sweden; Department of Surgery, Akademiska Sjukhuset, Uppsala, Sweden

**Keywords:** cholecystitis, cholangitis, pancreatitis, competing risk, retrospective cohort study

## Abstract

**Background:**

Non-operative management of gallstone complications (acute cholecystitis, cholangitis, and biliary pancreatitis) carries a significant risk of recurrence. Nevertheless, definitive surgery is often deferred in co-morbid patients due to their perceived short life expectancy. This study assessed the prevalence of non-operative management, and its associated mortality and recurrence risk, accounting for the competing risk of death due to other causes.

**Methods:**

A nationwide cohort of patients admitted with gallstone complications and treated with non-operative management between 2006 and 2019 was identified in the Swedish National Patient Register. Competing risk and Cox proportional hazards models were used to evaluate recurrence and mortality risks.

**Results:**

Overall, 82 101 patients were included (median age 68 years, 48% female), of whom 41 283 (50.3%) experienced cholecystitis, 10 272 (12.5%) experienced cholangitis, and 30 546 (37.2%) experienced pancreatitis. Patients with cholangitis were older and had more co-morbidities than those with cholecystitis or pancreatitis. The cumulative recurrence risk at 1 year was 16.0% for cholecystitis, cholangitis, and pancreatitis. At 3 years, the risk was 19.8% for cholecystitis and 23.0% for cholangitis and 22.5% for pancreatitis. In patients aged ≥ 80 years, 3-year mortality was 41.2%, and 17.0% of deaths were gallstone related. In competing risk analyses, cholangitis and pancreatitis were associated with a higher recurrence risk than cholecystitis in alive patients, with hazard ratio (HRs) of 1.22 (95% confidence interval (c.i.) 1.16 to 1.28) and 1.28 (95% c.i. 1.24 to 1.32), respectively, whereas female sex was associated with a lower recurrence risk (HR 0.92; 95% c.i. 0.89 to 0.94). Increasing age (HR 1.08; 95% c.i. 1.08 to 1.09), co-morbidities (HR 1.17; 95% c.i. 1.16 to 1.17), and cholangitis (HR 1.31; 95% c.i. 1.26 to 1.36) were associated with an increased risk of mortality.

**Conclusion:**

The risk of recurrent gallstone complications remains clinically important, even among older and co-morbid patients, despite their higher competing risk of death. These findings support consideration of early cholecystectomy in patients with an acceptable operative risk, with the aim of reducing subsequent morbidity and recurrence.

## Teaser

Non-operative management of gallstone complications is common in elderly and co-morbid patients, but carries a significant risk of recurrence despite high overall mortality. In this Swedish nationwide cohort of 82 101 patients, recurrence risk reached 21% at 3 years regardless of age, whereas 3-year mortality was 41% in those aged > 80 years, with 17% of mortality being gallstone related. Early cholecystectomy should be considered for more patients than current practice patterns suggest.

## Introduction

Acute cholangitis, cholecystitis, and pancreatitis are common complications in patients with cholelithiasis. Early cholecystectomy is the standard treatment for cholecystitis^1,2^, and is recommended for patients with cholangitis and biliary pancreatitis to reduce the risk of recurrent gallstone-related complications^3–5^. In patients with septic cholangitis, emergency cholecystectomy is associated with high mortality. In this scenario, endoscopic retrograde cholangiopancreatography (ERCP) is the recommended first-line intervention^6^, with interval cholecystectomy performed once the patient is no longer septic^4^. For patients with significant co-morbidities, ERCP is sometimes considered as an alternative treatment to cholecystectomy for gallstone-related complications, especially in patients with known common bile duct stones^7–9^.

Waiting for an elective cholecystectomy is associated with a risk of recurrence^10^, with recurrence occurring in approximately 30–40% of patients if no surgery is performed^11–15^. Several studies have demonstrated that mortality and costs are reduced if surgery is performed^14,16^, yet early surgery is not always offered to patients with complicated gallstone disease^12^. In light of studies indicating a potentially acceptable risk of severe postoperative complications and death even among patients with co-morbidities, early surgery may be considered in most patients if the risk of recurrence is sufficiently high^5,11,17^. However, some authors have argued that elderly and multimorbid patients may not survive long enough to experience recurrence, making conservative treatment a reasonable option in this population^18,19^. Previous studies have adjusted for this by censoring observations; however, it may be more appropriate to consider death as a competing risk, and analyse it accordingly.

This study aimed to investigate the competing risks of recurrences and mortality in patients discharged without undergoing cholecystectomy after an episode of cholecystitis, cholangitis, or biliary pancreatitis. In addition, the timing of and risk factors for recurrent complications of gallstone disease were investigated. Special attention was given to elderly patients (age ≥ 80 years), who are more likely to be co-morbid and frail, and have an increased risk of dying from other causes. The hypothesis was that the recurrence rate would be high and that many patients would survive long enough to experience recurrence before death.

## Methods

This study was approved by the Swedish Ethics Review Authority (dnr 2022-06449-01 and 2023-01971-01). The manuscript was prepared as per the STROBE guidelines^20^. This project was registered in 2022 as part of a bigger project in the Gävleborg County Institutional database (ID 279344) and with the Gallriks registry (ID 46586).

### Study population

This is a retrospective registry-based cohort study. Data were retrieved from the National Board of Health and Welfare’s National Patient Register (NPR), containing diagnostic and surgical codes for all hospitalizations in Sweden since 1987 and secondary care outpatient visits since 2001^21^. Patients aged > 18 years who had a discharge diagnosis, using the International Classification of Disease version 10 Swedish Edition (ICD-10-SE), of cholangitis (K80.3, K83.0), cholecystitis (K80.0, K80.1, K80.4, K81.0), or biliary pancreatitis (K85.1, K85.9) between 2006 and 2019 and were discharged without a surgical code for cholecystectomy were included. *Table S1* provides a detailed description of the codes. For the purpose of the analyses, patients diagnosed with both cholangitis and cholecystitis were classified as experiencing cholangitis; those with pancreatitis and other simultaneous complications were classified as experiencing pancreatitis. Patients who underwent cholecystectomy for cholangitis, cholecystitis, and/or biliary pancreatitis between 2006 and 2019 were identified using the Nordic Medico-Statistical Committee (NOMESCO) code for cholecystectomy (open or laparoscopic; JKA20, JKA21). ERCPs performed between 2006 and 2019 were identified through the NPR using the NOMESCO code UJK02^22^.

Patients who had a previous cholecystectomy, ERCP, or a gallstone complication between 2003 and 2006 in the NPR were excluded to remove recurrent gallstone complications using the above ICD-10-SE and NOMESCO codes. Procedures before 1997 were identified using the coding scheme used before 1997, with 535x (cholecystectomies) and the procedure 9014 (ERCP) being identified. Patients with a diagnosis of biliary, hepatic, or pancreatic cancer before or after their gallstone complication were identified using ICD-10-SE codes (C22–C25) and excluded. Patients who died before discharge were also excluded because they cannot experience a recurrence.

### Study variables and definitions

The exposures were cholecystitis, cholangitis, and biliary pancreatitis. The outcomes of interest were the recurrence of gallstone complications, surgery, and both all-cause and gallstone-related mortality.

The index event, defined as the first registered gallstone-related complication, was identified for each patient, and the time to recurrence, cholecystectomy, death, or end of follow-up was calculated. Death information was obtained from the National Cause of Death Register, an annually updated register covering all deaths occurring in Sweden, including those of individuals not residing in the country, with the purpose of describing causes of death and tracking mortality trends. Follow-up was closed on 31 December 2022, with data from the National Cause of Death Register ending on this date.

Recurrence was defined as an unplanned inpatient or emergency department visit at least 1 day after the discharge date of the previous hospitalization with at least one ICD-10-SE code of cholecystitis, cholangitis, or biliary pancreatitis. Outpatient clinic visits were not included in the calculation of recurrences, nor was planned surgery. The length of hospital stay for patients transferred between hospitals or wards was calculated as the time between the first admission and discharge from the last hospital by counting all in-patient stays with 0 days between discharge and admission as a continuous stay. Time between recurrent episodes was calculated if there was more than one recurrence, and the total length of stay over all these episodes was determined. The cause of death was defined as gallstone related if any cholecystitis, cholangitis, or biliary pancreatitis diagnosis (ICD-10-SE codes K80, K83 or K85) was included among the causes of death, and as caused by surgery if patients had a cholecystectomy 4 weeks before death, which is the same definition used by the National Cause of Death Register. If the exact day of death was unknown, it was encoded as occuring on the 15th day of the month of death.

Cholecystostomies were identified within the NPR using the codes JKA10–JKA13, whereas percutaneous transhepatic cholangiography with drainage (PTC) was identified using codes AJ022–AJ025; both these procedures were classified as non-operative treatment.

For the included patients, all inpatient and outpatient registrations in the NPR from 1964 up until their first admission for gallstone complications with ICD codes needed to calculate the Charlson co-morbidity index (CCI) were obtained^23,24^.

### Statistical analysis

No power analysis was performed because the aim of the study was to investigate all eligible patients. Missing data were reported and not imputed; in particular, multivariable analyses were conducted using complete case analysis; for example, excluding patients with missing values in any of the analysed variables. Tabulation by index event per patient, and by each complication was done. Separate tables were created for specific co-morbidities across the index event diagnosis. Follow-up was calculated using the reverse Kaplan–Meier estimator.

Life tables were calculated to facilitate comparisons between groups for complications and gallstone-related mortality. The log-rank test was used to compare survival between groups. Multivariable Cox proportional hazards regression analysis was used to assess the risk of recurrence adjusting for age, sex, CCI, type of initial complication, and whether ERCP was performed. Patients were censored for cholecystectomy, mortality, and loss to follow-up. To account for the competing risk of mortality, Aalen–Johansen estimators were calculated using a multivariable multistate competing risk regression model with the same adjustments as the Cox proportional hazards regression analyses above^25^. Death was coded as a competing risk and assessed in a multistate illness–death–recovery model where patients could move between a healthy state to recurrence or death or from recurrence back to a healthy state. This allows the estimation of event-specific hazard ratios (HRs) for covariates on the rate of transitions between states^26^. Data were transformed using the tmerge function, and the model was calculated using the coxph command^27,28^. Cumulative incidence curves were constructed according to diagnosis and stratified by age group to illustrate and test the differences in subdistribution hazards (Gray’s test) between groups using the cuminc command from the cmprsk package^29^.

A post hoc subgroup analysis of patients aged ≥ 80 years was performed to allow analysis of individuals with the highest risk of all-cause mortality. Statistical analyses were performed using R version 4.4.3 and R Studio 2024.12.1 (R Foundation for Statistical Computing, Vienna, Austria). The R packages cmprsk and survival were used to calculate the competing risk regressions and figures^27,29^.

## Results

There were 148 890 patients in the NPR with an inpatient registration of the relevant diagnostic codes. After excluding patients who had gallstone complications or surgery before 2006, surgery or ERCP before inpatient admission, a previous or future diagnosis of biliary, hepatic, or pancreatic cancer, and early surgery, there were 84 590 unique patients. Patients dying before discharge were then excluded, leaving 82 101 patients in the final cohort (*Fig. 1*).

**Figure 1:**
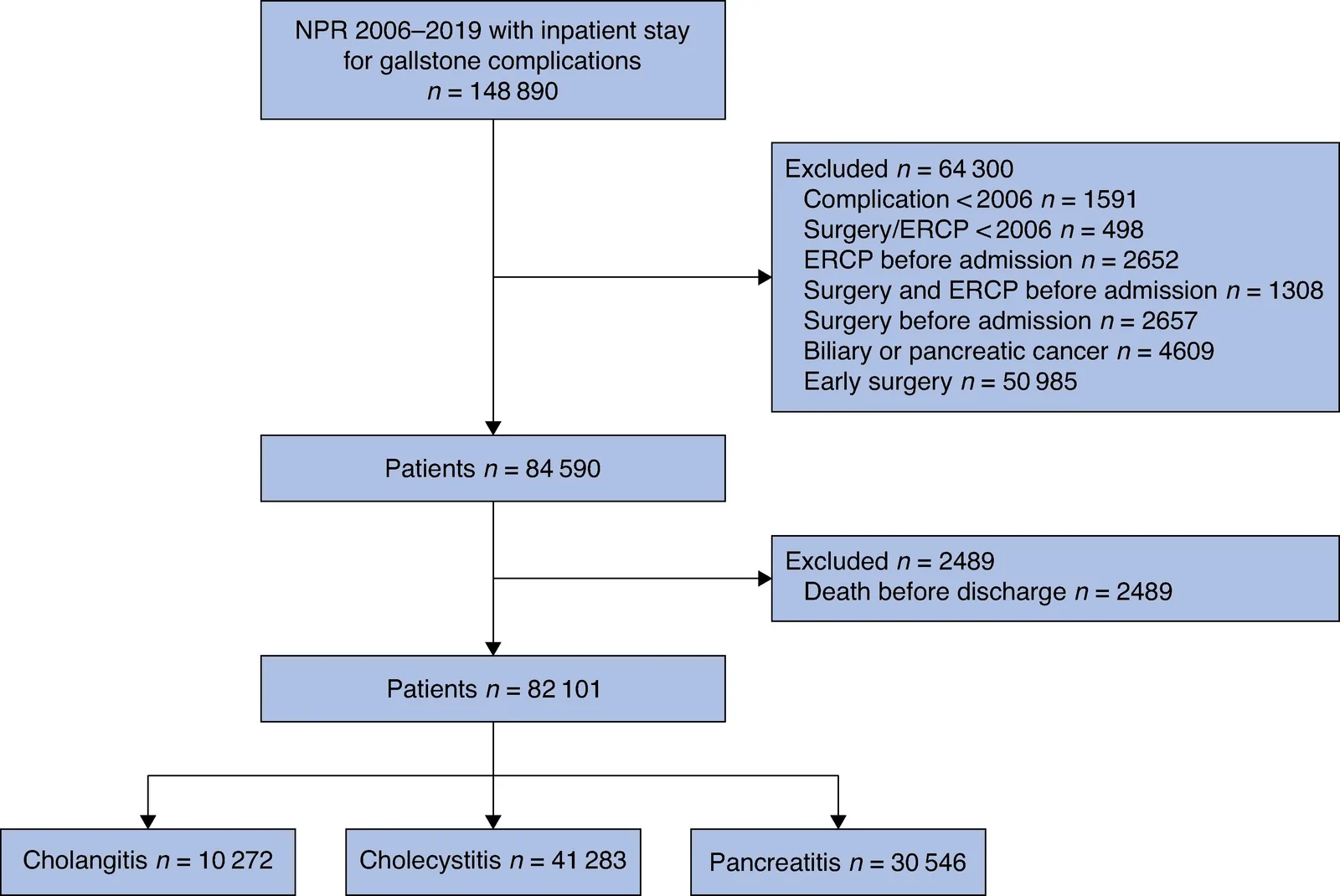
Flow chart of patient selection and health care contacts from the NPR 2006–2019 NPR, National Patient Register; ERCP, endoscopic retrograde cholangiopancreatography with sphincterotomy.

### Baseline characteristics of the study cohort

Overall, 79 562 patients experienced a single gallstone complication: cholecystitis, 41 283 (52.9%); pancreatitis, 28 539 (35.9%); and cholangitis, 9743 (12.2%). In addition, there were 529 patients with diagnoses of both cholangitis and cholecystitis, 587 patients with pancreatitis and cholangitis, 1352 patients with pancreatitis and cholecystitis, and 71 patients with all three diagnoses. After regrouping patients experiencing multiple complications, 41 283 patients (50.3%) were in the cholecystitis group, 30 546 patients (37.2%) were in the pancreatitis group, and 10 272 patients (12.5%) were in the cholangitis group.

The general characteristics and follow-up information of the study population stratified by index gallstone complication are presented in *Table 1*; the distribution of co-morbidities between groups is presented in *Table S2*. Patients with cholangitis were older, had more co-morbidities, and had a longer length of hospital stay than patients with cholecystitis and pancreatitis. Pre-existing liver diseases were rare in all groups. No PTC procedures were performed for the first event and only 64 cholecystostomies were performed (59 for cholecystitis, two for cholangitis, and three in patients with pancreatitis; *P* < 0.001). The median follow-up was 7.1 years overall, and 7.3, 7.6 and 4.7 years among cholecystitis, pancreatitis, and cholangitis patients, respectively. The characteristics of the healthcare contacts are presented in *Table S3*. Patients with cholangitis had a median of two inpatient stays related to gallstone complications, whereas those with cholecystitis and pancreatitis had a median of one in patient stay per patient.

**Table 1:** Demographic characteristics at baseline and follow-up data for the study population, stratified by index gallstone complication

	Overall (*n* = 82 101)	Cholecystitis (*n* = 41 283, 50%)	Pancreatitis (*n* = 30 546, 37%)	Cholangitis (*n* = 10 272, 13%)	P*
Age (years), median (i.q.r.)	68 (52–79)	69 (54–80)	63 (48–75)	78 (66–85)	< 0.001†
**Sex**					< 0.001
Male	42 408 (51.7%)	20 426 (49.5%)	16 843 (55.1%)	5139 (50.0%)	
Female	39 693 (48.3%)	20 857 (50.5%)	13 703 (44.9%)	5133 (50.0%)	
CCI, median (i.q.r.)	2 (0–4)	2 (0–4)	2 (0–4)	3 (1–5)	< 0.001†
**LOS (days), median (i.q.r.)**					
Index	4.0 (2–7)	4.0 (2–7)	4.0 (2–8)	5.0 (3–9)	< 0.001†
Total	6 (3–11)	5 (3–9)	6 (3–12)	8 (4–15)	< 0.001†
ERCP	8250 (10.0%)	1828 (4.4%)	2500 (8.2%)	3922 (38.2%)	< 0.001
PTC	0 (0.0%)	0 (0.0%)	0 (0.0%)	0 (0.0%)	
Cholecystostomy	64 (0.1%)	59 (0.1%)	3 (0.0%)	2 (0.0%)	< 0.001
**Recurrence**					< 0.001
None	56 682 (69.0%)	28 557 (69.2%)	21 040 (68.9%)	7085 (69.0%)	
Cholecystitis	13 136 (16.0%)	10 384 (25.2%)	1941 (6.4%)	811 (7.9%)	
Pancreatitis	8513 (10.4%)	1196 (2.9%)	7078 (23.2%)	239 (2.3%)	
Cholangitis	3770 (4.6%)	1146 (2.8%)	487 (1.6%)	2137 (20.8%)	
Surgery after complication	5607 (6.8%)	3398 (8.2%)	1988 (6.5%)	221 (2.2%)	< 0.001
Time to surgery (days), median, (iqr)	90 (45–176)	101 (58–183)	66 (29–150)	104 (48–248)	< 0.001†
Time to recurrence (days), median, (iqr)	99 (28–401)	90 (32–246)	112 (23–583)	149 (33–641)	
Dead within 30 days	1573 (1.9%)	704 (1.7%)	503 (1.6%)	366 (3.6%)	< 0.001†
Dead within 1 year	6814 (8.3%)	3253 (7.9%)	1969 (6.4%)	1592 (15.5%)	< 0.001†

Values are *n* (%) unless otherwise stated. There were no missing data. i.q.r., interquartile range; CCI, Charlson co-morbidity index; LOS, length of hospital stay; ERCP, endoscopic retrograde cholangiopancreatography with sphincterotomy; PTC, percutaneus transhepatic cholangiography. *χ^2^ test, except †Kruskal–Wallis test.

### Recurrence and survival

The overall recurrence rate was 31.0% (25 419 of 82 101) and was similar in the three groups. Recurrences usually came in the form of the same gallstone-related complication as the index event (*Table 1*). The frequency of cholecystectomy after discharge for the index event was 6.8% in the overall population, but higher in patients with cholecystitis and pancreatitis relative to those with cholangitis. Patients with pancreatitis had a shorter time from discharge to surgery. Both 30-day and 1-year mortality were higher among patients with cholangitis.

The risks of mortality and recurrence at various time points are presented in *Table 2* for the overall cohort and in *Table S4* for the subset of patients over 80 years of age. Kaplan–Meier curves for the cumulative incidence of recurrence stratified by initial diagnosis are shown in *Fig. S1* for the overall population and in *Fig. S2* for patients aged over 80 years. The cumulative recurrence risk at 1 year was 16.0% for cholecystitis, cholangitis, and pancreatitis. At 3 years, the risk was 19.8% for cholecystitis, 23.0% for cholangitis, and 22.5% for pancreatitis (*Table 2*). The yearly recurrence rate decreased after 2 years, and few recurrences occurred after 3 years. The 3-year all-cause mortality was 16.6% after cholecystitis, 30.3% after cholangitis, and 12.8% after pancreatitis in the overall population (*P* < 0.001). In patients aged over 80 years, 3-year all-cause mortality was 40.6%, 45.4%, and 38.6% after cholecystitis, cholangitis, and pancreatitis, respectively (*P* < 0.001). The 3-year gallstone complication-specific mortality was 2.0% after cholecystitis, 4.5% after cholangitis, and 2.5% after pancreatitis in all patients. In individuals over 80 years of age, 3-year gallstone complication-specific mortality was 6.3%, 7.2%, and 8.1% after cholecystitis, cholangitis, and pancreatitis, respectively, accounting for 16.6% of all mortality in that age group (*Table S4*).

**Table 2:** Risk of overall and gallstone complication-related mortality and risk of recurrence at various time points, censoring for surgery, death, and follow-up (only first event included)

Outcome	Index diagnosis	Time point					*P*
		1 year	2 years	3 years	4 years	5 years	
All-cause mortality	Overall	8.3 (8.1, 8.5)	12.9 (12.7, 13.1)	16.9 (16.7, 17.2)	20.8 (20.5, 21.1)	24.3 (24.0, 24.6)	
	Cholecystitis	7.9 (7.6, 8.1)	12.6 (12.3, 12.9)	16.6 (16.3, 17)	20.7 (20.3, 21.1)	24.2 (23.8, 24.6)	< 0.001
	Pancreatitis	6.4 (6.2, 6.7)	9.8 (9.5, 10.2)	12.8 (12.4, 13.2)	15.7 (15.3, 16.1)	18.5 (18.0, 18.9)	
	Cholangitis	15.5 (14.8, 16.2)	23.3 (22.4, 24.1)	30.3 (29.4, 31.1)	36.6 (35.7, 37.5)	42.1 (41.1, 43.1)	
Gallstone complication-related mortality	Overall	1.8 (1.7, 1.9)	2.2 (2.1, 2.3)	2.5 (2.4, 2.6)	2.7 (2.6, 2.8)	2.9 (2.8, 3.0)	
	Cholecystitis	1.5 (1.4, 1.6)	1.8 (1.7, 2.0)	2.0 (1.9, 2.2)	2.2 (2.1, 2.4)	2.3 (2.2, 2.5)	< 0.001
	Pancreatitis	1.8 (1.7, 2.0)	2.2 (2.0, 2.3)	2.5 (2.3, 2.6)	2.7 (2.5, 2.8)	2.8 (2.6, 3.0)	
	Cholangitis	3.2 (2.8, 3.5)	3.9 (3.5, 4.3)	4.5 (4.1, 4.9)	5.0 (4.5, 5.4)	5.3 (4.9, 5.8)	
Recurrences	Overall	16.0 (15.7, 16.2)	19.2 (18.9, 19.5)	21.3 (21, 21.6)	22.8 (22.4, 23.1)	23.9 (23.6, 24.2)	
	Cholecystitis	15.7 (15.3, 16.1)	18.1 (17.7, 18.5)	19.8 (19.4, 20.2)	20.9 (20.5, 21.4)	21.9 (21.4, 22.3)	< 0.001
	Pancreatitis	16.4 (16.0, 16.8)	20.2 (19.7, 20.7)	22.5 (22.0, 23.0)	24.3 (23.7, 24.8)	25.5 (24.9, 26.0)	
	Cholangitis	15.5 (14.8, 16.3)	19.9 (19.0, 20.7)	23.0 (22.1, 23.9)	25.0 (24.1, 26.0)	26.8 (25.8, 27.8)	

Values in parentheses are 95% confidence intervals. *P* values are from log-rank tests for differences in survival. Data for the subgroup aged ≥ 80 years are available in *Table S4*.

### Patients undergoing ERCP

There were 8250 patients who had an ERCP upon their index admission; their characteristics and outcomes by initial diagnosis are presented in *Table S5*. ERCP was more frequently performed in patients with cholangitis (3922 of 10 272, 38.2%) than in patients with cholecystitis (1828 of 41 283, 4.4%) or pancreatitis (2500 of 30 546, 8.2%; *P* < 0.001). Patients undergoing ERCP had longer initial lengths of hospital stay than those not undergoing ERCP: 7 *versus* 4 days (*P* < 0.001) for cholecystitis and pancreatitis, and 5 *versus* 6 days (*P* < 0.001) for cholangitis. Mortality at 30 days and 1 year was lower in patients with cholangitis who underwent ERCP than in those who did not (2.7% *versus* 4.1% and 12.5% *versus* 17.4%, respectively; both *P* < 0.001), whereas there were no significant differences in mortality based on the receipt of ERCP among patients with cholecystitis or pancreatitis. Time to first recurrence was longer in patients who underwent ERCP after cholecystitis and cholangitis, whereas there was no difference for pancreatitis (*Table S5*).

### Risk factors for recurrence

Cox proportional hazards models were calculated to investigate risk factors for recurrence (*Table 3*). In the overall population, male sex, increasing age, CCI, and a diagnosis of cholangitis or pancreatitis were independently associated with increased risk of recurrence. In the subset of patients aged ≥ 80 years, sex, CCI, and pancreatitis remained independently associated with recurrence. When stratifying the analysis by initial diagnosis, ERCP was associated with an increased risk of recurrence in patients with pancreatitis.

**Table 3:** Multivariable Cox proportional hazards regression for risk factors for new complications, censoring for death, surgery, and follow-up

	Hazard ratio	*P*	Hazard ratio	*P*	Hazard ratio	*P*
	All patients (*n* = 82 101)		Age <80 years (*n* = 61 794)		Age ≥80 years (*n* = 20 307)	
Age (years)	1.00 (1.00, 1.00)	< 0.001	1.00 (1.00, 1.00)	0.050	1.00 (0.99, 1.01)	0.794
Female *versus* male	0.93 (0.90, 0.96)	< 0.001	0.92 (0.89, 0.95)	< 0.001	0.94 (0.88, 1.00)	0.037
CCI (cont.)	1.03 (1.02, 1.04)	< 0.001	1.03 (1.02, 1.04)	< 0.001	1.03 (1.01, 1.04)	< 0.001
Cholangitis *versus* cholecystitis	1.17 (1.11, 1.23)	< 0.001	1.28 (1.20, 1.36)	< 0.001	1.01 (0.93, 1.10)	0.728
Pancreatitis *versus* cholecystitis	1.21 (1.18, 1.25)	< 0.001	1.24 (1.19, 1.28)	< 0.001	1.18 (1.10, 1.27)	< 0.001
ERCP *versus* no ERCP	1.03 (0.98, 1.09)	0.228	1.10 (1.03, 1.18)	0.003	0.92 (0.84, 1.00)	0.057

Per diagnosis	Cholecystitis (*n* = 41 283)		Cholangitis (*n* = 10 272)		Pancreatitis (*n* = 30 546)	
Age (years)	1.01 (1.01, 1.01)	< 0.001	1.00 (0.99, 1.00)	< 0.001	1.00 (1.00, 1.00)	0.024
Female *versus* male	0.93 (0.89, 0.98)	0.024	0.92 (0.85, 1.00)	0.040	0.94 (0.90, 0.99)	0.013
CCI (cont.)	1.02 (1.01, 1.03)	< 0.001	1.05 (1.04, 1.07)	< 0.001	1.03 (1.02, 1.04)	< 0.001
ERCP *versus* no ERCP	1.02 (0.92, 1.15)	0.671	0.92 (0.84, 1.00)	0.063	1.21 (1.12, 1.32)	< 0.001

Values in parentheses are 95% confidence intervals. CCI, Charlson co-morbidity index; cont., modelled as continuous variable; ERCP, endoscopic retrograde cholangiopancreatography with sphincterotomy.

### Competing risk analysis

To account for the competing risk of death from other causes, competing risk regressions were calculated (*Table 4*). Cholangitis and pancreatitis were associated with an elevated risk of recurrence than cholecystitis. Cholangitis was associated with a higher risk of death than cholecystitis, whereas there was no significant difference in the risk of death between cholecystitis and pancreatitis. Women had a lower risk of recurrence and death; age and co-morbidities were correlated with both recurrence and mortality, and the HRs were larger for mortality, indicating a larger effect on mortality than recurrence. Patients who underwent ERCP had a lower risk of mortality.

**Table 4:** Competing risk regression for the risk of recurrence or death, censoring for loss to follow-up, surgery- and gallstone-related deaths, and other causes of death

Outcome	Covariate	Hazard ratio	*P*
Competing risk of recurrence and death (*n* = 82 101 and *n* = 61 211 events)			
Risk of recurrence	Cholangitis *versus* cholecystitis	1.22 (1.16, 1.28)	< 0.001
	Pancreatitis *versus* cholecystitis	1.28 (1.24, 1.32)	< 0.001
	Female *versus* male	0.92 (0.89, 0.94)	< 0.001
	Age (years)	1.00 (1.00, 1.01)	< 0.001
	CCI (Cont.)	1.04 (1.04, 1.05)	< 0.001
	ERCP *versus* no ERCP	1.02 (0.97, 1.08)	0.477
Risk of death	Cholangitis *versus* cholecystitis	1.31 (1.26, 1.36)	< 0.001
	Pancreatitis *versus* cholecystitis	1.03 (1.00, 1.06)	0.084
	Female *versus* male	0.93 (0.91, 0.95)	< 0.001
	Age (years)	1.08 (1.08, 1.09)	< 0.001
	CCI (Cont.)	1.17 (1.16, 1.17)	< 0.001
	ERCP *versus* no ERCP	0.82 (0.78, 0.85)	< 0.001

Competing risk of gallstone-related death *versus* other death (*n* = 82 101 and *n* = 32 316 events)			
Risk of gallstone-related death	Cholangitis *versus* cholecystitis	1.76 (1.57, 1.96)	< 0.001
	Pancreatitis *versus* cholecystitis	1.82 (1.67, 1.99)	< 0.001
	Female *versus* male	1.03 (0.95, 1.11)	0.471
	Age (years)	1.07 (1.07, 1.08)	< 0.001
	CCI (cont.)	1.04 (1.02, 1.05)	< 0.001
	ERCP *versus* no ERCP	0.71 (0.63, 0.81)	< 0.001
Risk of other death	Cholangitis *versus* cholecystitis	1.32 (1.27, 1.36)	< 0.001
	Pancreatitis *versus* cholecystitis	1.00 (0.97, 1.02)	0.771
	Female *versus* male	0.93 (0.91, 0.95)	< 0.001
	Age (years)	1.09 (1.08, 1.09)	< 0.001
	CCI (cont.)	1.17 (1.17, 1.18)	< 0.001
	ERCP *versus* no ERCP	0.84 (0.81, 0.87)	< 0.001

Values in parentheses are 95% confidence intervals. Initial complication compared to cholecystitis. CCI, Charlson co-morbidity index; cont., modelled as continuous variable; ERCP, endoscopic retrograde cholangiopancreatography with sphincterotomy.

Cumulative incidence curves of recurrence and mortality for all patients and for those aged ≥ 80 years are presented in *Figs 2* and *3*. The cumulative incidence curve for mortality crossed that for recurrence for patients aged ≥ 80 years with cholangitis at 2 years and cholecystitis at 3 years, whereas it did not cross for pancreatitis within 5 years. Competing risk curves of recurrence and death after the first complication with cholangitis and pancreatitis according to ERCP status for patients aged ≥ 80 years are presented in *Figs S3* and *S4*. Competing risk curves of gallstone-related death *versus* other death after the first complication are shown in *Fig. S5*, with no crossover of curves, but with a significant difference between diagnoses (Grays test *P* < 0.001. The risk of gallstone-related deaths was higher in patients with cholangitis and pancreatitis, as well as in elderly and co-morbid patients. ERCP was associated with a lower risk of gallstone-related deaths and death from other causes (*Table 4*).

**Figure 2:**
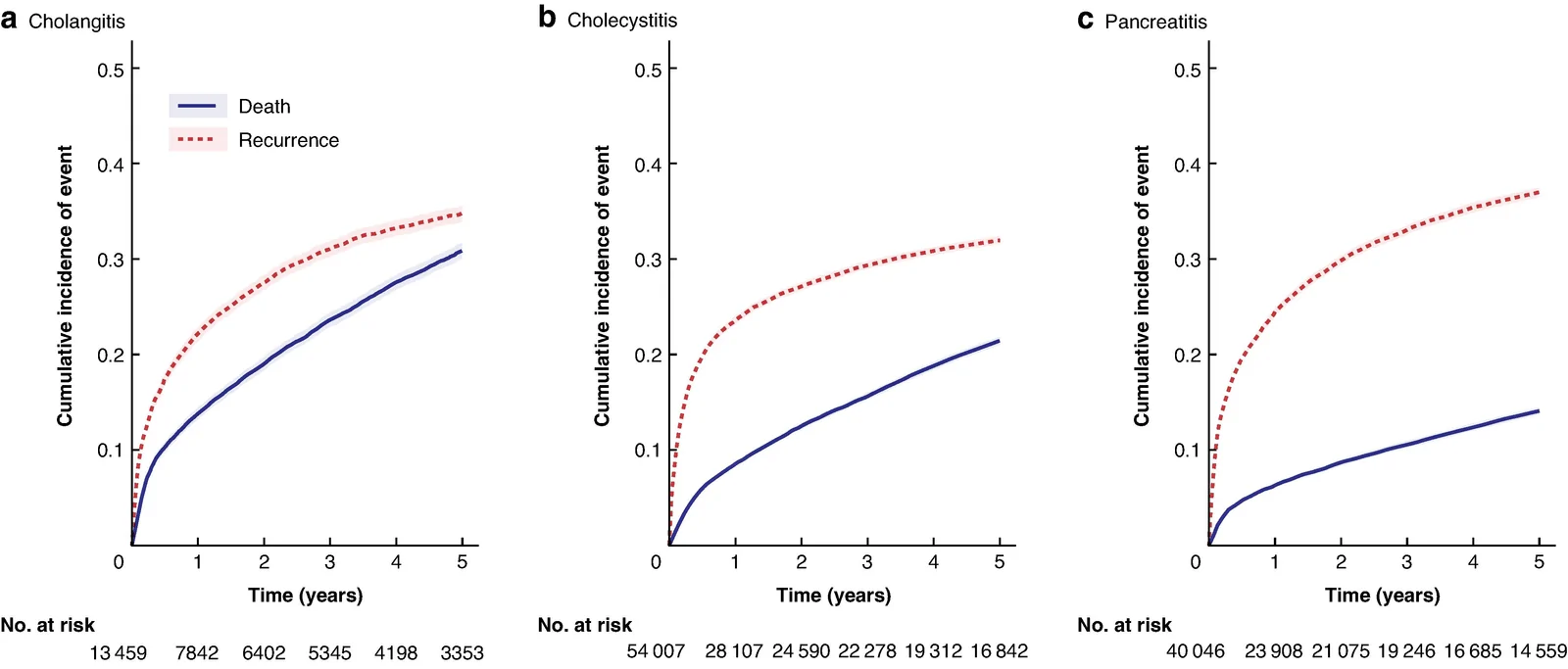
Competing risk analysis of death and recurrence risk, censoring for end of follow-up or surgery after non-operative management of cholecystitis, cholangitis, and pancreatitis for all patients The significance of differences between diagnoses of **a** cholecystitis, **b** cholangitis, and **c** pancreatitis for each outcome was tested with Gray’s test for differences of subdistribution hazards (*P* < 0.001 for both outcomes). Shaded areas indicate 95% confidence intervals.

**Figure 3:**
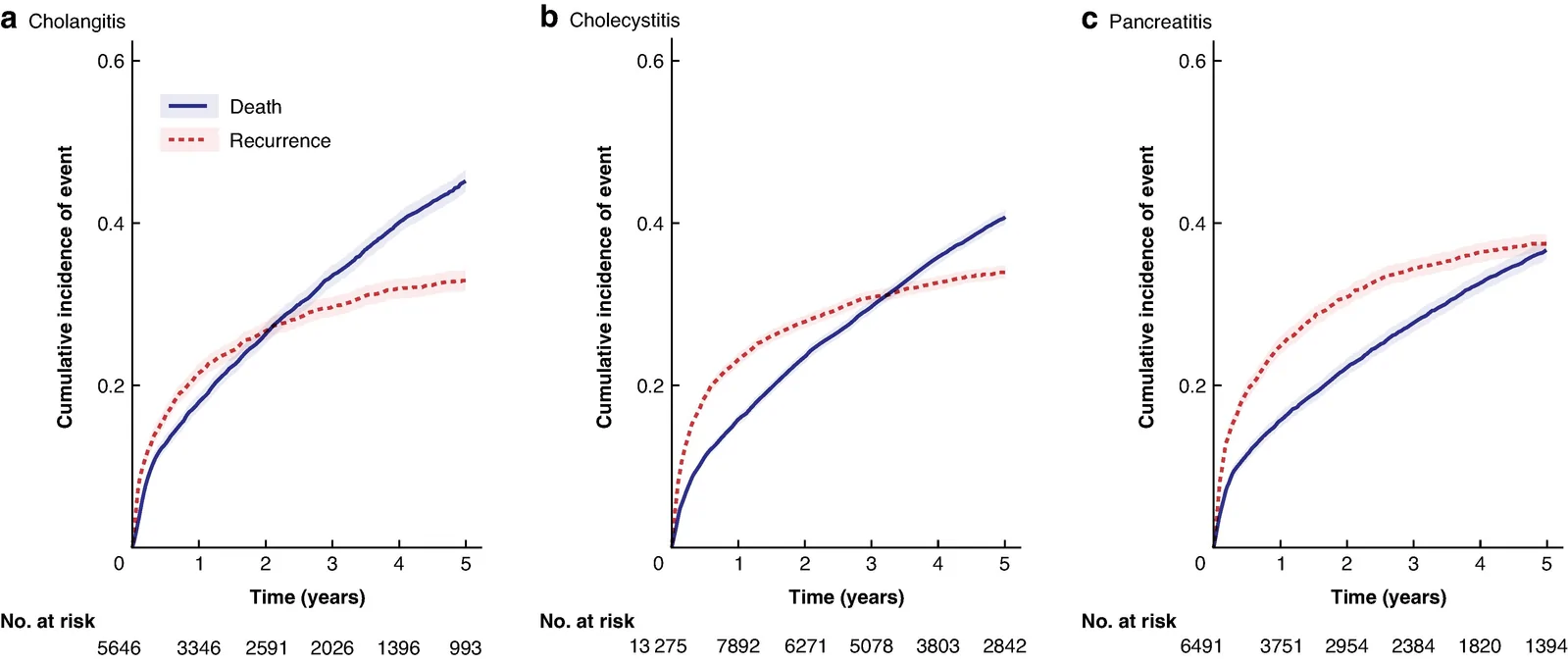
Competing risk analysis of death and recurrence risk, censoring for end of follow-up or surgery after non-operative management of cholecystitis, cholangitis, and pancreatitis for patients age ≥ 80 years in the Swedish National Patient Register 2006–2019 The significance of differences between diagnoses of **a** cholecystitis, **b** cholangitis, and **c** pancreatitis for each outcome was tested with Gray’s test for differences of subdistribution hazards (*P* < 0.001 for both outcomes). Shaded areas indicate 95% confidence intervals.

## Discussion

In this nationwide cohort of patients with non-operative management of gallstone complications (cholecystitis, cholangitis, pancreatitis), the recurrence rate was high across all three diagnoses, regardless of patient age. Even when accounting for the competing risk of death, the risk of recurrence remained high, and patients experienced recurrence regardless of age or co-morbidities. Recurrences came early, with one in five patients experiencing recurrence the first 3 years, after which the rate decreased. Cholangitis and pancreatitis were associated with a higher recurrence rate than cholecystitis. Elderly patients had a higher risk of mortality; despite this, many patients experienced recurrence before dying from other causes, and 17% of deaths had recurrent gallstone complications as a contributing cause.

The general population is ageing, but as the general health of the population and treatment for many diseases improve, the population eligible for cholecystectomy after gallstone complications will increase. Here, two models of recurrence risk were used: a regular Cox proportional hazards regression and a competing risk regression. The Cox regression assumes patients were immortal, whereas the competing risk regression takes mortality into account. In patients discharged without cholecystectomy after gallstone complications, the mortality risk was 24.3% at 5 years, with a 23.9% recurrence risk. These figures increased to 58.8% and 27.3% at 5 years, respectively, in the subset of patients aged ≥ 80 years. Many recurrent gallstone complications, some of which were fatal, could therefore have been prevented by performing a cholecystectomy at the index event. These results support the trend towards early surgery to reduce recurrence, morbidity, and healthcare resource utilization, because many patients treated with non-operative management survived long enough to experience recurrence. Notably, a significant proportion of patients who did not receive early surgery were young and had no co-morbidities; hopefully, future evaluations will show an increased uptake of surgery in this population, because patients with an expected long-term survival after discharge represent ideal candidates for cholecystectomy^2^. Previous studies have shown that cholecystectomy prevents recurrent biliary pancreatitis, but the optimal timing for surgery after moderate and severe pancreatitis remains debated^3^. In a previous study^11^, cholecystectomy for cholecystitis resulted in postoperative complications in 25–50% of patients with severe co-morbidities, yet mortality and costs were lower in the early cholecystectomy group^13,16^. A recent trial^30^ of cholecystectomy for cholecystitis had trouble recruiting enough elderly and frail patients to meet targets. A study evaluating hospital practice patterns in Sweden^31^ found that hospitals treating a higher proportion of patients aged over 70 years with cholecystectomy for cholecystitis had shorter overall lengths of hospital stay, with no difference in mortality. According to previous literature^5,17^, early cholecystectomy after cholangitis and pancreatitis is associated with a lower risk of complications than for cholecystitis. ERCP could be considered for patients not undergoing surgery for cholangitis and pancreatitis, but it does not prevent recurrence in cholecystitis, and the duration of its protective effect in patients with pancreatitis is unknown^8,32,33^. Further research is needed on the complication risks and optimal timing of surgery for cholangitis, as well as the effect of ERCP *versus* surgery or no intervention in elderly patients with pancreatitis. The recurrence risk observed in this study was similar to risks reported in other international analyses^11–15^. In a previous study on common bile duct stones, only 11% of patients over 75 years of age experienced recurrence; in the present cohort, using a narrower definition of older age, 27% of patients with cholangitis experienced recurrence within 5 years^9^. Contrary to previous studies^15^ suggesting that recurrences tend to occur 1 year or later after non-operative management of cholecystitis, in the present study one-quarter of recurrences occurred within 30 days and half occurred within 100 days.

The strengths of this study include accounting for death as a competing risk and the nationwide coverage across Sweden. Despite long-standing recommendations for early cholecystectomy in patients with cholecystitis and pancreatitis, a large cohort in which the disease was allowed to follow its natural course was identified and analysed. This study has significant limitations, primarily related to its registry-based design. In particular, the definition of gallstone-related mortality based on ICD-10-SE codes in death certificates is pragmatic but potentially imprecise. The National Cause of Death register has been demonstrated to be reliable for in-hospital deaths, whereas out-of-hospital deaths appear to be less covered.^34,35^ Although the data covered all new gallstone complications between 2006 and 2019 with follow-up until 2023, a broad definition of gallstone diseases was chosen to cover coding variation between hospitals. In addition, patients with the most severe disease likely died before discharge and were excluded, there was no measure of disease severity available for risk-adjustment, and there may be missing data for cholecystostomies and PTC procedures, because these were likely used in more than 0.1% of patients. The method to calculate the CCI has been used in previous studies and is considered to give representative results but does not account for patient frailty and all concomitant medications^23,36^. Moreover, the results regarding ERCP should be interpreted cautiously. In this study, ERCP was likely used in select patients who were diagnosed or suspected of having common bile duct stones and/or biliary duct obstruction in addition to cholecystitis, cholangitis, or pancreatitis, which represents a more severe form of the disease. The three gallstone-related complications analysed are not equal because cholangitis and pancreatitis have a higher baseline mortality relative to cholecystitis, so that a reduction of recurrence in these patients may be of greater importance. Finally, worldwide differences in healthcare systems and the availability of operative resources may limit the generalizability of the present results.

## Supplementary Material

zrag149_Supplementary_Data

## Data Availability

The data that support the findings of this study are available from the Swedish National Board of Welfare. Restrictions apply to the availability of these data, which were used under licence for this study.
