# Supplementary material for: Recurrence and mortality after non-operative management of acute gallstone disease: nationwide registry study
Source: BJS Open. 2026 Nov 4;10(6):zrag149. doi: 10.1093/bjsopen/zrag149 (PMC13732573; doi:10.1093/bjsopen/zrag149)
Supplement: Supplementary file 1 [file zrag149_supplementary_data.docx]

**Recurrence and mortality after non-operative management of acute gallstone disease: nationwide registry study**

Maria Söderström^1,2^, Olov Norlén^1,2^ ,Fredrik Linder^1,2^, Erik Osterman^1,2^

^1^Department of Surgical Sciences, Uppsala University, Uppsala, Sweden
^2^Department of Surgery, Akademiska Sjukhuset, Uppsala, Sweden

**Corresponding author:** Erik Osterman Region Uppsala, Akademiska Sjukhuset, Department of Surgery, 751 85 Uppsala Sweden

[Erik.osterman@uu.se](mailto:Erik.osterman@uu.se) **ORCID-ID 0000-0003-1621-7872**

**Supplementary Materials – Index**

| **Supplementary Appendixes** |  |
| --- | --- |
| Strobe checklist | 2 |
| **Supplementary Figures and Tables** |  |
| Supplementary Figure S1 | 4 |
| Supplementary Figure S2 | 5 |
| Supplementary Figure S3 | 6 |
| Supplementary Figure S4 | 7 |
| Supplemenatry Figure S5 | 8 |
| Supplementary Table S1 | 9 |
| Supplementary Table S2 | 10 |
| Supplementary Table S3 | 11 |
| Supplementary Table S4 | 12 |
| Supplementary Table S5 | 13 |

# **Supplementary Appendices**

## **Strobe checklist**

|  | Item No | Recommendation | Page  No |
| --- | --- | --- | --- |
| **Title and abstract** | 1 | (*a*) Indicate the study’s design with a commonly used term in the title or the abstract | 1 and 2 (abstract) |
|  |  | (*b*) Provide in the abstract an informative and balanced summary of what was done and what was found | Abstract |
| Introduction | | | |
| Background/rationale | 2 | Explain the scientific background and rationale for the investigation being reported | 4 |
| Objectives | 3 | State specific objectives, including any prespecified hypotheses | 4+5 |
| Methods | | | |
| Study design | 4 | Present key elements of study design early in the paper | 6 |
| Setting | 5 | Describe the setting, locations, and relevant dates, including periods of recruitment, exposure, follow-up, and data collection | 7 |
| Participants | 6 | (*a*) *Cohort study*—Give the eligibility criteria, and the sources and methods of selection of participants. Describe methods of follow-up | 6+7, Supplementary Table 1 |
|  |  | (*b*) *Cohort study*—For matched studies, give matching criteria and number of exposed and unexposed  *Case-control study*—For matched studies, give matching criteria and the number of controls per case | NA |
| Variables | 7 | Clearly define all outcomes, exposures, predictors, potential confounders, and effect modifiers. Give diagnostic criteria, if applicable | 6-8 |
| Data sources/ measurement | 8* | For each variable of interest, give sources of data and details of methods of assessment (measurement). Describe comparability of assessment methods if there is more than one group | *6-8* |
| Bias | 9 | Describe any efforts to address potential sources of bias | Subgroups analysis on page 8 and 9 |
| Study size | 10 | Explain how the study size was arrived at | 8 |
| Quantitative variables | 11 | Explain how quantitative variables were handled in the analyses. If applicable, describe which groupings were chosen and why | 7-8 |
| Statistical methods | 12 | (*a*) Describe all statistical methods, including those used to control for confounding | 8-9 |
|  |  | (*b*) Describe any methods used to examine subgroups and interactions | 8-9 |
|  |  | (*c*) Explain how missing data were addressed | 8 |
|  |  | (*d*) *Cohort study*—If applicable, explain how loss to follow-up was addressed | 7 |
|  |  | (*e*) Describe any sensitivity analyses | NA |

Continued on next page

| Results | | | |
| --- | --- | --- | --- |
| Participants | 13* | (a) Report numbers of individuals at each stage of study—eg numbers potentially eligible, examined for eligibility, confirmed eligible, included in the study, completing follow-up, and analysed | 10 |
|  |  | (b) Give reasons for non-participation at each stage | 10 |
|  |  | (c) Consider use of a flow diagram | Figure 1 |
| Descriptive data | 14* | (a) Give characteristics of study participants (eg demographic, clinical, social) and information on exposures and potential confounders | Table 1, Supplementary Table 2-3 and 5 |
|  |  | (b) Indicate number of participants with missing data for each variable of interest | Table 1 (no missing data) |
|  |  | (c) *Cohort study*—Summarise follow-up time (eg, average and total amount) | Page 10 for follow-up, Table 1 time to events in patients experiencing events |
| Outcome data | 15* | *Cohort study*—Report numbers of outcome events or summary measures over time | *Table 1 and Table 2* |
| Main results | 16 | (*a*) Give unadjusted estimates and, if applicable, confounder-adjusted estimates and their precision (eg, 95% confidence interval). Make clear which confounders were adjusted for and why they were included | Tables 2-4, Supplementary tables 4 and page 10-13 |
|  |  | (*b*) Report category boundaries when continuous variables were categorized | NA |
|  |  | (*c*) If relevant, consider translating estimates of relative risk into absolute risk for a meaningful time period | NA |
| Other analyses | 17 | Report other analyses done—eg analyses of subgroups and interactions, and sensitivity analyses | 10-13 (Age ≥80 reported continuously) |
| Discussion | | | |
| Key results | 18 | Summarise key results with reference to study objectives | 14 |
| 11Limitations | 19 | Discuss limitations of the study, taking into account sources of potential bias or imprecision. Discuss both direction and magnitude of any potential bias | 16 |
| Interpretation | 20 | Give a cautious overall interpretation of results considering objectives, limitations, multiplicity of analyses, results from similar studies, and other relevant evidence | 14 |
| Generalisability | 21 | Discuss the generalisability (external validity) of the study results | 16 |
| Other information | | | |
| Funding | 22 | Give the source of funding and the role of the funders for the present study and, if applicable, for the original study on which the present article is based | 1 |

**Supplementary Figure S1: Kaplan Meier curve of recurrences after first complication from gallstones. Number at risk table below graph.** Vertical lines represent censored patients. Shaded area is the 95% Confidence interval

ALT-TEXT: Event over time curve. Recurrences rise fastest during the first 2 years and then flattens out. Recurrences after cholecystitis and pancreatitis are more common than cholangitis.

**Supplementary Figure S2: Kaplan Meier curve of recurrence for patients 80 and over by initial complication.** Vertical lines represent censored patients. Shaded area is the 95% Confidence interval

ALT-TEXT: Event over time curve for patients over 80. Recurrences rise fastest during the first 2 years and then flattens out. Recurrences after cholecystitis are more common than cholangitis and pancreatitis.

No ERCP

ERCP

**Supplementary Figure S3: Competing risk curve of recurrences and death after the first complication with cholangitis by ERCP for patients ≥80 years of age.** Lines for the cumulative incidence of events. Green lines mortality and orange lines recurrences. Shaded area is the 95% confidence interval. Time measured in years. Gray’s test 0.028 for recurrences and 0.009 for mortality.

ALT-TEXT: Events over time graph showing death and recurrences for patients with cholangitis by ERCP or not. Mortality increase from 0 to around 45% while recurrences rise to 35% over 5 years. The curves look similair with and without ERCP.

No ERCP

ERCP

**Supplementary figure S4: Competing risk curve of recurrences and death after the first complication with pancreatitis by ERCP for patients ≥80 years of age.** Lines for the cumulative incidence of events. Green lines mortality and orange lines recurrences. Shaded area is the 95% confidence interval. Time measured in years. Gray’s test 0.027 for recurrences and <0.001 for mortality.

ALT-TEXT: Events over time graph showing death and recurrences for patients with pancreatitis by ERCP or not. Recurrences increase from 0 to around 35% while mortlity reach the same number without ERCP but significantly lower with ERCP over 5 years.

**Supplementary figure S5: Competing risk curve of gallstone related death and other death after the first complication.** Difference between diagnoses for each outcome were tested with Gray’s test for differences of subdistribution hazards. Lines for the cumulative incidence of events. Blue lines (GD) gallstone related death, green lines other death. Shaded area is the 95% confidence interval. Time measured in years.

ALT-TEXT: Events over time graph showing death and gallstone related deaths as competing risks. Death increases from 0 to around 30-40% for Cholangitis, cholecystitis and pancreatitis over 5 years. Gallstone related deaths increases from 0 to 5-10% over the same time.

**Supplementary Table S1: Coding scheme for identification of patients**

| **Diagnosis** | **ICD-10 SE** | **Meaning/Interpretation** |
| --- | --- | --- |
| **Cholangitis** | **K803 K830** | Choledocolithiasis with cholangitis Cholangitis |
| **Cholecystitis** | **K800 K801 K804 K810** | Cholecystolithiasis with cholecystitis Choledocolithiasis with other cholecystitis Choledocolithiasis with cholecystitis Acute Cholecystitis |
| **Pancreatitis** | **K851 K859** | Acute biliary pancreatitis Acute pancreatitis, unspecified |
| **Hepato-Biliary and Pancreatic cancers** | **C22 C23 C24 C25** | Hepatic cancers Gallbladder cancer Biliary cancer Pancreatic cancer |
| **Surgery/Intervention** | **NOMESCO code** | **Meaning/Interpretation** |
| **Cholecystectomy** | **JKA2x 535x** | Cholecystectomy open/laparoscopic Cholecystectomy |
| **ERCP** | **UJKxx 9014** | ERCP ± biopsy/cholangioscopy ERCP |
| **Cholecystostomy** | **JKA1x** | Cholecystostomy open/laparoscopic/percutaneus |
| **Percutaneus transhepatic drain** | **AJ022 AJ023 AJ024 AJ025** | Cholangiography, PTC and external drain Cholangiography, PTC dilation/removal of stone Cholangiography, PTC Cholangiography, PTC, internal drain |

**ERCP=Endoscopic retrograde cholangiopancreatography; PTC=Percutaneus transhepatic cholangiography**

**Supplementary Table S2: Comorbidities of the study cohort stratified by initial complication.**

|  | **Overall** | **Cholecystitis** | **Pancreatitis** | **Cholangitis** | **p** |
| --- | --- | --- | --- | --- | --- |
| **Characteristic** | N = 82,101 | N = 41,283 | N = 30,546 | N = 10,272 |  |
| Myocardial infarction | 12,190 (14.8%) | 6,413 (15.5%) | 3,895 (12.8%) | 1,882 (18.3%) | <0.001 |
| Congestive heart failure | 18,374 (22.4%) | 9,605 (23.3%) | 5,564 (18.2%) | 3,205 (31.2%) | <0.001 |
| Peripheral vascular disease | 8,306 (10.1%) | 4,172 (10.1%) | 2,779 (9.1%) | 1,355 (13.2%) | <0.001 |
| Cerebrovascular disease | 17,381 (21.2%) | 9,276 (22.5%) | 5,348 (17.5%) | 2,757 (26.8%) | <0.001 |
| Chronic obstructive pulmonary disease | 8,371 (10.2%) | 4,160 (10.1%) | 2,938 (9.6%) | 1,273 (12.4%) | <0.001 |
| Chronic other pulmonary disease | 9,860 (12.0%) | 4,999 (12.1%) | 3,558 (11.6%) | 1,303 (12.7%) | 0.014 |
| Rheumatic disease | 7,427 (9.0%) | 3,685 (8.9%) | 2,517 (8.2%) | 1,225 (11.9%) | <0.001 |
| Dementia | 7,280 (8.9%) | 4,004 (9.7%) | 1,966 (6.4%) | 1,310 (12.8%) | <0.001 |
| Hemiplegia | 1,177 (1.4%) | 607 (1.5%) | 409 (1.3%) | 161 (1.6%) | 0.163 |
| Diabetes without chronic complication | 630 (0.8%) | 264 (0.6%) | 284 (0.9%) | 82 (0.8%) | <0.001 |
| Diabetes with chronic complication | 6,689 (8.1%) | 3,364 (8.1%) | 2,329 (7.6%) | 996 (9.7%) | <0.001 |
| Renal disease | 9,342 (11.4%) | 4,611 (11.2%) | 3,119 (10.2%) | 1,612 (15.7%) | <0.001 |
| Mild liver disease | 2,071 (2.5%) | 822 (2.0%) | 898 (2.9%) | 351 (3.4%) | <0.001 |
| Severe liver disease | 1,394 (1.7%) | 417 (1.0%) | 591 (1.9%) | 386 (3.8%) | <0.001 |
| Peptic ulcer disease | 7,572 (9.2%) | 3,480 (8.4%) | 2,851 (9.3%) | 1,241 (12.1%) | <0.001 |
| Malignancy | 19,594 (23.9%) | 10,118 (24.5%) | 6,452 (21.1%) | 3,024 (29.4%) | <0.001 |
| Metastatic solid tumour | 5,438 (6.6%) | 2,676 (6.5%) | 1,825 (6.0%) | 937 (9.1%) | <0.001 |
| Aids | 176 (0.2%) | 72 (0.2%) | 91 (0.3%) | 13 (0.1%) | <0.001 |

P values computed with the chi-square test

**Supplementary table S3: Characteristics of cohort by event instead of per patient.** Each patient could experience several gallstone complications during the course of the study. This table shows the characteristics of 82,101 patients during the course of the study and their 140,849 gallstone complications.

| **Characteristic** | **Overall**  N = 140,849 | **Cholecystitis**  N = 65,489 | **Pancreatitis**  N = 51,518 | **Cholangitis**  N = 23,842 | **p** |
| --- | --- | --- | --- | --- | --- |
| In patient stays for gallstone complications (count) | 1 (1, 2) | 1 (1, 2) | 1 (1, 2) | 2 (1, 3) | <0.001 |
| Age (Years) | 68 (52, 80) | 70 (55, 80) | 63 (48, 76) | 75 (57, 84) | <0.001 |
| Sex |  |  |  |  | <0.001 |
| Male | 74,774 (53.1%) | 32,987 (50.4%) | 28,977 (56.2%) | 12,810 (53.7%) |  |
| Female | 66,075 (46.9%) | 32,502 (49.6%) | 22,541 (43.8%) | 11,032 (46.3%) |  |
| CCI | 2 (0, 4) | 2 (0, 4) | 2 (0, 4) | 3 (1, 5) | <0.001 |
| LoS (Days) | 4 (2, 7) | 4 (2, 7) | 4 (2, 8) | 5(3, 9) | <0.001 |
| ERCP | 15,031 (10.7%) | 3,253 (5.0%) | 4,243 (8.2%) | 7,535 (31.6%) | <0.001 |
| PTC | 3 (0.0%) | 0 (0.0%) | 0 (0.0%) | 3 (0.0%) | * |
| Cholecystostomy | 557 (0.4%) | 502 (0.8%) | 27 (0.1%) | 28 (0.1%) | <0.001 |
| Recurrence |  |  |  |  | <0.001 |
| None | 95,156 (67.6%) | 47,489 (72.5%) | 33,551 (65.1%) | 14,116 (59.2%) |  |
| Cholecystitis | 18,337 (13.0%) | 14,107 (21.5%) | 2,843 (5.5%) | 1,387 (5.8%) |  |
| Cholangitis | 16,516 (11.7%) | 1,857 (2.8%) | 14,129 (27.4%) | 530 (2.2%) |  |
| Pancreatitis | 10,840 (7.7%) | 2,036 (3.1%) | 995 (1.9%) | 7,809 (32.8%) |  |
| Time to recurrence (Days) | 97 (26, 378) | 89 (30, 270) | 112 (23, 491) | 98 (25, 397) | <0.001 |
| Dead within 30 days | 3,811 (2.7%) | 1,411 (2.2%) | 1,302 (2.5%) | 1,098 (4.6%) | <0.001 |
| Dead within 1 year | 13,099 (9.3%) | 5,415 (8.3%) | 3,915 (7.6%) | 3,769 (15.8%) | <0.001 |

CCI: Charlson Comorbidity Index

ERCP: Endoscopic retrograde cholangiopancreatography with sphincterotomy
PTC: Percutaneous transhepatic drain

LoS: Length of Stays
* Can not be tested since PTC were only done in one group.

**Supplementary Table S4: Risk of overall and gallstone complication-related mortality and risk of recurrence for patients ≥80 at various time points, censoring for surgery, death and follow-up (only first event included).**

| **Outcome** | **Index diagnosis** | **Time 1y**  **% (95%CI)** | **Time 2y**  **% (95%CI)** | **Time 3y**  **% (95%CI)** | **Time 4y**  **% (95%CI)** | **Time 5y**  **% (95%CI)** | **p** |
| --- | --- | --- | --- | --- | --- | --- | --- |
| All-cause mortality  ≥80 | Overall | 20.0(19.4, 20.5) | 31.4(30.8, 32.0) | 41.2(40.5, 41.9) | 50.8(50.1, 51.5) | 58.8(58.1, 59.5) |  |
|  | Cholecystitis | 19.6(18.9, 20.4) | 31(30.1, 31.8) | 40.6(39.7, 41.5) | 50.3(49.3, 51.2) | 58.2(57.2, 59.1) | <0.001 |
|  | Pancreatitis | 19.3(18.2, 20.4) | 29.7(28.4, 30.9) | 38.6(37.3, 40.0) | 47.3(45.9, 48.7) | 54.8(53.4, 56.2) |  |
|  | Cholangitis | 21.7(20.4, 22.8) | 34.3(32.9, 35.7) | 45.4(43.9, 46.8) | 55.9(54.4, 57.3) | 64.5(63.1, 65.9) |  |
| Gallstone complication-related mortality  ≥80 | Overall | 5.0 (4.7, 5.3) | 6.1 (5.7, 6.4) | 7.0 (6.6, 7.4) | 7.6 (7.2, 8.0) | 8.2 (7.8, 8.7) |  |
|  | Cholecystitis | 4.6 (4.2, 5.0) | 5.6 (5.1, 6.0) | 6.3 (5.9, 6.8) | 6.9 (6.4, 7.5) | 7.4 (6.9, 8.0) | <0.001 |
|  | Pancreatitis | 5.7 (5.0, 6.3) | 6.9 (6.2, 7.7) | 8.1 (7.3, 8.9) | 8.8 (7.9, 9.6) | 9.4 (8.5, 10.3) |  |
|  | Cholangitis | 5.2 (4.5, 5.8) | 6.3 (5.5, 7.0) | 7.2 (6.4, 8.0) | 8.0 (7.1, 8.9) | 8.9 (7.9, 9.8) |  |
| Recurrences  ≥80 | Overall | 16.0(15.5, 16.6) | 20.3(19.7, 20.9) | 23.3(22.6, 23.9) | 25.5(24.8, 26.2) | 27.3(26.5, 28.0) |  |
|  | Cholecystitis | 15.8(15.1, 16.5) | 19.6(18.8, 20.4) | 22.5(21.6, 23.3) | 24.5(23.5, 25.4) | 26.4(25.4, 27.4) | <0.001 |
|  | Pancreatitis | 17.7(16.5, 18.8) | 22.7(21.5, 24.0) | 26.1(24.7, 27.4) | 28.3(26.9, 29.7) | 29.8(28.3, 31.3) |  |
|  | Cholangitis | 14.8(13.7, 15.9) | 19.2(17.9, 20.4) | 22.2(20.8, 23.5) | 24.9(23.4, 26.3) | 26.6(25.0, 28.2) |  |

95%CI: 95% Confidence interval of estimate
p: p-value of the log-rank test for differences between survival
Y: years

**Supplementary Table S5: Characteristics of patients by ERCP at the index admission**

| **Cholecystitis** | **Overall**  N=41,283 | **No ERCP**  N=39,455 | **ERCP**  N=1,828 | **p** |
| --- | --- | --- | --- | --- |
| Age (Years) | 69 (54, 80) | 69 (53, 80) | 74 (61, 83) | <0.001 |
| Sex |  |  |  | 0.073 |
| Male | 20,426 (49.5%) | 19,559 (49.6%) | 867 (47.4%) |  |
| Female | 20,857 (50.5%) | 19,896 (50.4%) | 961 (52.6%) |  |
| CCI | 2 (0, 4) | 2 (0, 4) | 2 (1, 4) | <0.001 |
| LoS (Days) | 4(2, 7) | 4(2, 6) | 7 (4, 11) | <0.001 |
| Recurrence |  |  |  | <0.001 |
| None | 28,557 (69.2%) | 27,179 (68.9%) | 1,378 (75.4%) |  |
| Cholecystitis | 10,384 (25.2%) | 10,047 (25.5%) | 337 (18.4%) |  |
| Pancreatitis | 1,196 (2.9%) | 1,175 (3.0%) | 21 (1.1%) |  |
| Cholangitis | 1,146 (2.8%) | 1,054 (2.7%) | 92 (5.0%) |  |
| Time to recurrence (Days) | 90 (32, 246) | 89 (32, 240) | 123 (38, 504) | <0.001 |
| Dead within 30 days | 704 (1.7%) | 681 (1.7%) | 23 (1.3%) | 0.131 |
| Dead within 1 year | 3,253 (7.9%) | 3,109 (7.9%) | 144 (7.9%) | 0.997 |
| **Cholangitis** | **Overall**  N= 10,272 | **No ERPC**  N= 6,350 | **ERCP**  N= 3,922 | **p** |
| Age (Years) | 78 (66, 85) | 76 (63, 84) | 80 (69, 86) | <0.001 |
| Sex |  |  |  | 0.719 |
| Male | 5,139 (50.0%) | 3,168 (49.9%) | 1,971 (50.3%) |  |
| Female | 5,133 (50.0%) | 3,182 (50.1%) | 1,951 (49.7%) |  |
| CCI | 3 (1, 5) | 3 (1, 5) | 3 (1, 5) | 0.488 |
| LoS (Days) | 5 (3, 9) | 5 (3, 8) | 6 (4, 10) | <0.001 |
| Recurrence |  |  |  | <0.001 |
| None | 7,085 (69.0%) | 4,250 (66.9%) | 2,835 (72.3%) |  |
| Cholecystitis | 811 (7.9%) | 452 (7.1%) | 359 (9.2%) |  |
| Pancreatitis | 239 (2.3%) | 190 (3.0%) | 49 (1.2%) |  |
| Cholangitis | 2,137 (20.8%) | 1,458 (23.0%) | 679 (17.3%) |  |
| Time to recurrence (Days) | 149 (33, 641) | 137 (29, 632) | 170 (40, 661) | 0.024 |
| Dead within 30 days | 366 (3.6%) | 262 (4.1%) | 104 (2.7%) | <0.001 |
| Dead within 1 year | 1,592 (15.5%) | 1,102 (17.4%) | 490 (12.5%) | <0.001 |
| **Pancreatitis** | **Overall**  N =30,546 | **No ERCP**  N=28,046 | **ERCP**  N=2,500 | **p** |
| Age (Years) | 63 (48, 75) | 62 (47, 74) | 71 (57, 81) | <0.001 |
| Sex |  |  |  | <0.001 |
| Male | 16,843 (55.1%) | 15,617 (55.7%) | 1,226 (49.0%) |  |
| Female | 13,703 (44.9%) | 12,429 (44.3%) | 1,274 (51.0%) |  |
| CCI | 2 (0, 4) | 2 (0, 4) | 2 (0, 4) | <0.001 |
| LoS (Days) | 4 (2, 8) | 4 (2, 7) | 7 (5, 13) | <0.001 |
| Recurrence |  |  |  | <0.001 |
| None | 21,040 (68.9%) | 19,407 (69.2%) | 1,633 (65.3%) |  |
| Cholecystitis | 1,941 (6.4%) | 1,513 (5.4%) | 428 (17.1%) |  |
| Pancreatitis | 7,078 (23.2%) | 6,790 (24.2%) | 288 (11.5%) |  |
| Cholangitis | 487 (1.6%) | 336 (1.2%) | 151 (6.0%) |  |
| Time to recurrence (Days) | 112 (23, 583) | 113 (22, 587) | 105 (28, 525) | <0.971 |
| Dead within 30 days | 503 (1.6%) | 467 (1.7%) | 36 (1.4%) | 0.397 |
| Dead within 1 year | 1,969 (6.4%) | 1,798 (6.4%) | 171 (6.8%) | 0.402 |

CCI: Charlson Comorbidity Index, LoS: Length of Stays
